# Evaluating Facelift Complications and the Effectiveness of the SMASectomy Technique: A Single Center’s 15-Year Experience

**DOI:** 10.1093/asjof/ojab030

**Published:** 2021-08-20

**Authors:** Orr Shauly, Gregory L Stone, Rebeca Shin, W Grant Stevens, Daniel J Gould

## Abstract

**Background:**

Facelift continues to be one of the most common aesthetic procedures performed in the United States. Although there exist many techniques and variations, superficial musculoaponeurotic system (SMAS) manipulation, by way of plication, overlap, or SMASectomy, is common and has been shown to result in favorable cosmesis and durability. However, there is a lack of current complications data in the discussion of this technique.

**Objectives:**

To assess the benefits and risks of the SMASectomy technique.

**Methods:**

The records of all patients who underwent a facelift procedure between December 2004 and March 2019 were reviewed for this study. All procedures were performed at an American Association for Accreditation of Ambulatory Surgery Facilities (AAAASF)-accredited outpatient facility in Marina Del Rey, California. This represents data on 241 total patients. Retrospective chart review was performed to include data on patient characteristics, operative technique, and complications.

**Results:**

Average operative time of 152.68 ± 51.50 minutes and anesthesia time of 175.00 ± 54.07 minutes were observed among those patients who underwent SMASectomy. This was significantly lower (*P* < 0.000001) than those who did not undergo SMASectomy (average operative time of 265.25 ± 85.25 minutes and anesthesia time of 294.22 ± 85.31 minutes). There were no observed facial nerve injuries among patients who underwent SMASectomy. No deep vein thrombosis (DVT) events were observed in this patient population.

**Conclusions:**

In the hands of an experienced surgeon, the SMASectomy facelift technique offers the unique advantage of significantly reducing operating time and anesthesia time and can provide extremely favorable and long-lasting aesthetic results.

**Level of Evidence: 3:**

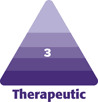

Facelift has maintained its position as the seventh-most common cosmetic surgical procedure worldwide for more than 2 decades and is the most common aesthetic procedure performed in patients over the age of 65. According to The Aesthetic Society’s National Data Bank statistics of 2019, there was an increase of more than 6% in the total surgical procedures performed since 2015. Even so, facelift surgery has seen an almost 8% downturn in this time. The majority of these procedures have been performed on women (94%), between the ages of 35 and 50 (40%).^1^ Despite this setback, few other surgical procedures have seen as much innovation and change over the years as the rhytidectomy, with many new refinements in technique even over the past decade.^2-6^ However, the decrease in demand is likely multifactorial in nature with the perception of unnatural outcomes likely having influence on this trend, as well as the relative lack of experienced providers to keep up with the demand.^7^

The rhytidectomy procedure evolved from the subcutaneous flap originally described in the early 20th century. The superficial flap soon gave way to the deeper fascial layer manipulation that we know today, with a hallmark study by Skoog first describing the dissection of the deep fascial layers in 1960.^8^ A better understanding of the deeper layers of the face was then established by Mitz and Peyronie in their important cadaveric study that first described the superficial musculoaponeurotic system (SMAS).^9^ They noted that this layer was continuous with the platysma, the temporoparietal fascia, and enveloped the facial muscles, protecting the vasculature and facial nerves. The discovery of this distinct facial layer paved the way for modern facelift techniques, many of which are still popular today.^10-18^

With this greater understanding, techniques developed that suspend the SMAS in order to remove tension from the overlying skin and enable optimal healing of facial incisions and to increase the longevity of the facelift aesthetic outcome.^19-23^ Many studies have shown support of this theory, with documentation of a much greater improvement in facial rejuvenation and longevity of outcomes.^24-28^ Even so, others have shown that deeper manipulation does not improve these results, and many surgeons in fact avoid SMAS dissection for the fear of facial nerve injury.

There are many other medical considerations when performing a facelift, which include perioperative and anesthetic considerations in addition to the surgical technique used as discussed above. Although a plethora of accepted techniques exists for facelift surgery, there remains significant individual surgeon variation leading to inconclusive data on outcomes. Even so, large single-center studies such as that by Abboushi et al in 2012 have attempted to document these complications and highlight the added risk of DVT.^29^ Published statistics since this time have continued to underscore the DVT risk in facelift procedure; however, many of these are derived from surveys of members of the American Society of Plastic Surgeons (ASPS), which suffers from response bias and low reliability of self-reported data.^3,30,31^

In light of these concerns, and a lack of current complications data, we examined the operative outcomes of facelift procedures performed at a private outpatient center to determine overall complication rates as well as those of SMAS manipulating procedures compared with less aggressive approaches. In addition, we evaluated the overall DVT risk at our institution and methods we took to mitigate this risk. We also sought to examine the additional perioperative benefits of SMAS dissection, including decreased anesthesia time and overall operative time, which may decrease patient risk factors of DVT and improve overall outcomes.

## METHODS

The objective of this study is to provide a comprehensive evaluation of complications associated with facelift procedures and to delineate the specific risks and benefits of SMAS manipulation in our practice. This study adhered to the guidelines set forth by the Declaration of Helsinki. Inclusion criteria were defined as those patients who underwent comprehensive preoperative workup and extensive follow-up of at least 60-days postoperatively. Patients who were lost to follow-up were excluded from this study. Informed consent was provided by all patients in this study. 

### Patients

The records of all patients who underwent a facelift procedure between December 2004 and March 2019 were retrospectively reviewed for this study. All procedures were performed at our AAAASF-accredited outpatient facility in Marina Del Rey, California. This represents data on 241 total patients. Facelifts were performed by 2 surgeons at the practice who use similar techniques, identical preoperative and postoperative care, and similar management algorithms.

Chart review was performed, and data were inputted into an excel document with parameters including demographic data—gender, age, body mass index (BMI), and clinical data—past surgical history, comorbidities, tobacco use, steroid use, hormone replacement therapy use, diabetes, hypertension, and documented history of easy bleeding. In addition, extensive operative data were collected, including primary vs secondary facelift, additional procedures performed at the time of facelift (unrelated to the facelift itself—abdominoplasty, breast augmentation, and other cosmetic procedures), total number of past facelifts, Decadron given, steroids prescribed, facial drains placed, operative time, anesthesia time, Caprini score, postoperative laser use, and follow-up time. Operative time was the total time spent on the table for all procedures performed from skin incision to skin closure. Additional operative data were collected that included facelift technique used and liposuction or fat grafting volume. All patients underwent comprehensive preoperative workup including a full history and physical examination, laboratory work, and Electrocardiogram (EKG) for clearance by anesthesia. 

All patients were administered general anesthesia by an anesthesiologist, and given the estimated operative time, DVT prophylaxis was limited to compression stockings and sequential compression devices. All patients were monitored overnight before discharge. All postoperative complications occurring within at least 30 days and 60 days were recorded for all patients, including return to operating room (OR) (facelift related). Complications were defined as any adverse postoperative event that was a direct consequence of the facelift or related facial procedure.

### Operative Technique

This study represents data on those patients who underwent a SMASectomy technique facelift (*n* = 189) and non-SMASectomy technique (*n* = 52). In the non-SMASectomy cohort, face plication was the primary technique used and, in some cases, SMAS flap was also used. Face plication was performed in 42 patients who did not undergo the SMASectomy technique, and in one patient in conjunction with SMASectomy. Additionally, SMAS flaps were performed in 5 patients.

**Figure 1.**
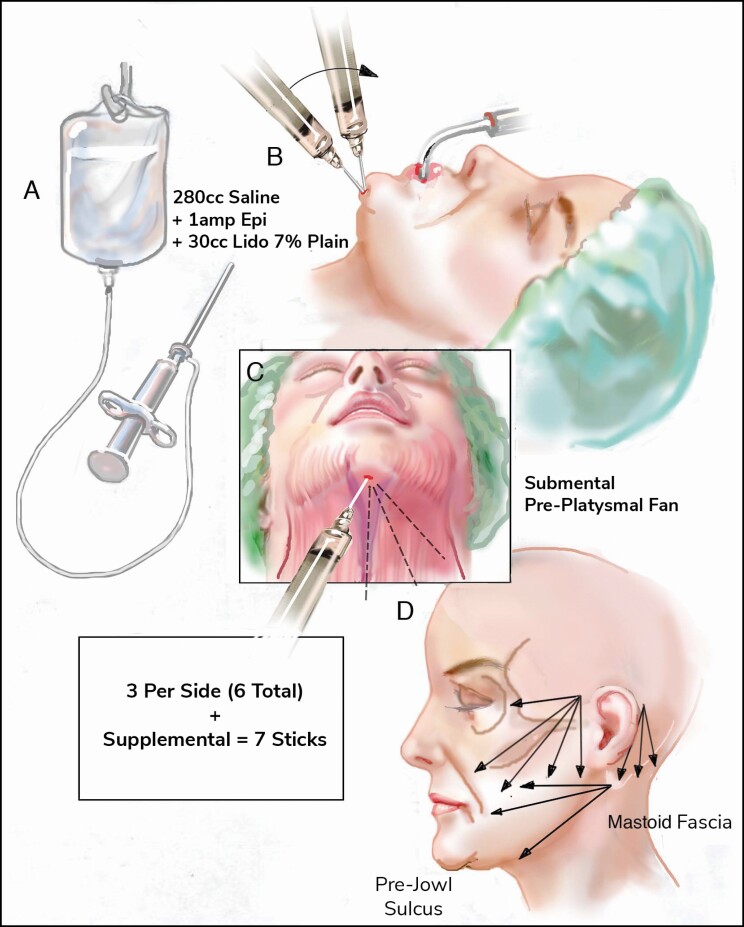
Illustration of Steven’s Tumescent technique demonstrating (A) the use of 750 mL saline tumescent solution containing 1 amp epinephrine and 30 mL 7% lidocaine plane, (B) injection technique, (C) injection locations, and (D) distribution of tumescent solution across the face following injection.

An extremely important factor in facelift surgery is the timing and technique of injection. In this practice, the facelift injection involves the use of a 280-mL bag of premixed tumescent solution containing 250-mL saline with 1 amp of epinephrine and 20 mL of 1% lidocaine solution. The bag is attached to a controlled delivery syringe and a spinal needle, with a 1-way valve so that the syringe can be easily auto reloaded. The tumescent is delivered first into the submental incision and deep throughout the neck in the preplatysmal plane, past the markings of the hyoid and lateral to the border of the external jugular vein. Then, the right side is injected from 3 points—one in front of the crus of the helix, to deliver to the cheeks and as far down on the nasolabial fold as possible, then from the base of the lobe to reach the neck and as far medial at the mandibular retaining ligament, and from behind the ear at the apex of the skin incision to hydrodissect the retroauricular space and the skin off of the mastoid fascia all the way posterior along the hairline and as far as the trapezius if needed in extremely lax necks. The left side is subsequently injected, and this is all done before prepping and draping the patient to allow time for efficacy. In this practice, we call this process the tumescence dissection technique, as the hydrodissection aids in facelift dissection significantly (Figure 1).

**Figure 2.**
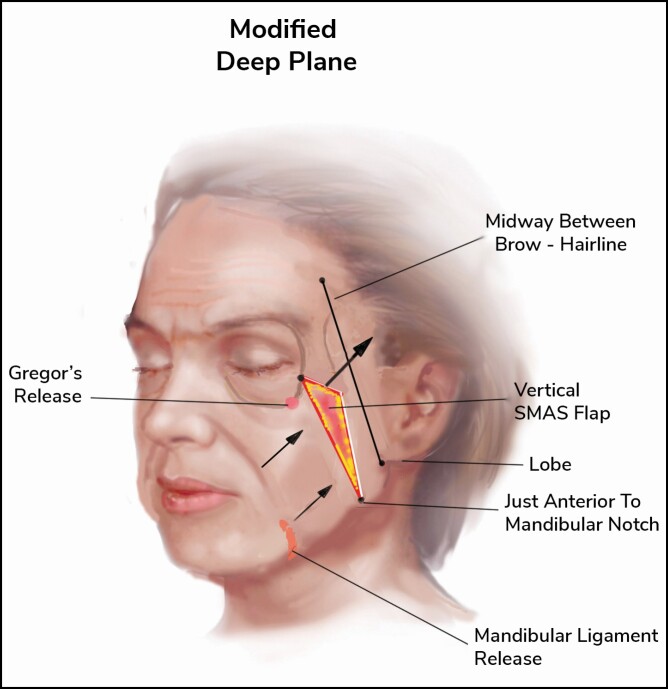
Modified deep-plane technique, demonstrating the vertical SMAS flap that will be elevated with release of the mandibular ligament. SMAS, superficial musculoaponeurotic system.

Skin incisions are equally essential as they help to camouflage the signs of facelift, though each incision is customized based on the amount of dissection and skin which needs to come from the neck and midface. The senior surgeon prefers to avoid an anterior incision if possible and will sometimes extend the incisions superior into the hairline when extreme vertical facelift is required. Retrotragal incisions are almost always used unless there is a deep pretragal sulcus, with care to avoid the region anterior to the hairline if possible. This can be predicted by palpation—if the cheek requires more than 2.5 cm of vertical movement and the face is mobile enough to permit it, then consider a vertical incision or an anterior incision which wraps around the hairline forward. If the neck likewise has extreme laxity and on pull there is more than 3 cm of lateral laxity, then consider an incision that either extends back into the hairline at the level of the auricular muscle or extend the incision posterior along the hairline past the lobe and even down into the low hairline if needed. It is important to remember that tension creates and exacerbates scars, so a complete release avoids a stretched or obvious scar regardless of placement in the hairline or along the hairline (Figure 2).

**Figure 3.**
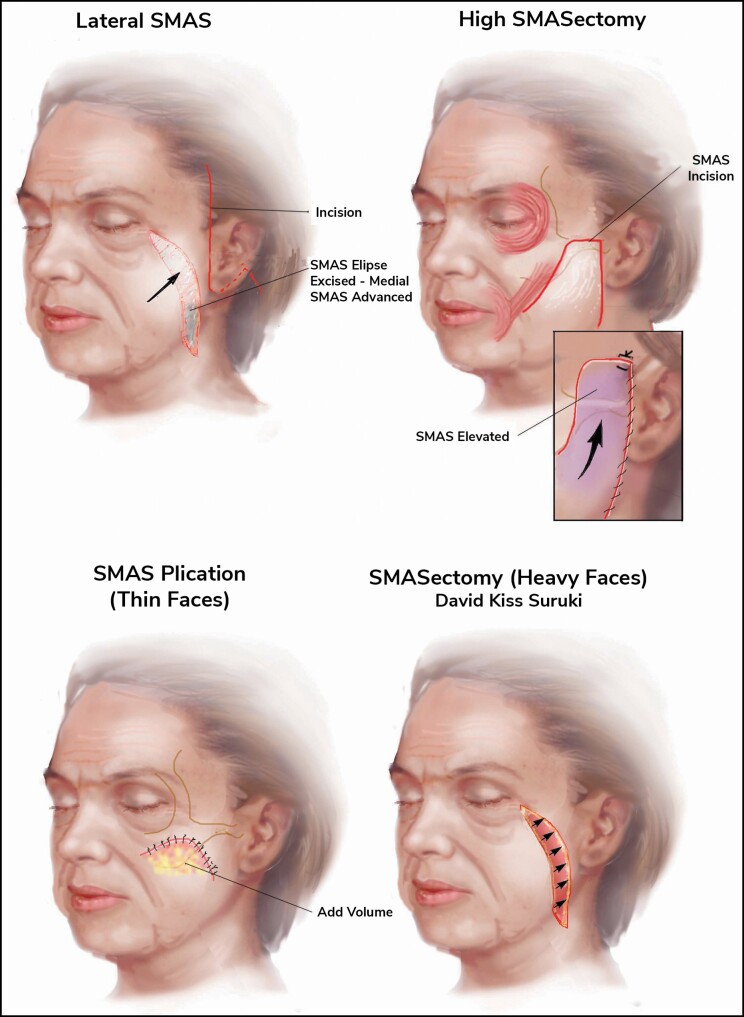
Depiction of various facelift techniques, with top left panel illustration of the lateral SMAS technique as seen by the lateral incision made here; top right panel illustration of the high SMASectomy technique, with the incision modified to allow for SMAS elevation; bottom left panel demonstrating the SMAS plication technique often used in thinner faces; and the SMASectomy technique incision modified for heavy faces. SMAS, superficial musculoaponeurotic system.

**Figure 4.**
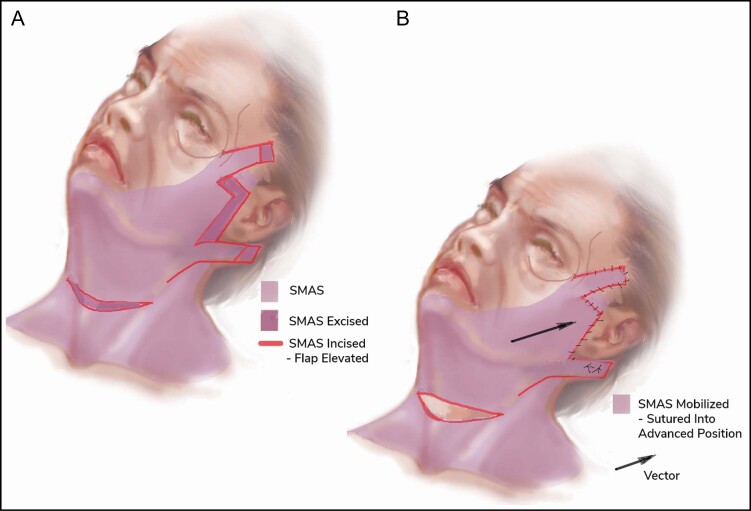
(A, B) Illustration of the SMAS layer of the face and areas of incision and subsequent excision in order to mobilize the SMAS and provide adequate tension. SMAS, superficial musculoaponeurotic system.

This practice utilizes several operative techniques and customizes them to match the needs of each patient, with heavier faces needing a greater extent of SMASectomy, whereas thinner patients may need fat grafting or SMAS overlapping or plication in some cases (Figure 3). The SMASectomy technique is ever-evolving, and in this practice, a lateral or high SMASectomy is often used for the favorable cosmetic results and longevity. It has also been recently documented in the literature that deep plane and composite extended SMASectomy have been more common in practice.^32,33^ Over time, the senior surgeon in this practice developed a technique largely based on SMASectomy as a cornerstone, with additional fat grafting or plication when needed in different cases (Figure 4). It is important to note that in this study, patients who only underwent SMAS plication or overlap were not included in the SMASectomy cohort.

The SMASectomy technique can vary in several ways. The modified deep plane is the newest approach that is still in development and combines the deep plane with lateral subplatysmal dissection. This technique leaves skin attached to the SMAS flap, thus providing a great result with very minimal swelling. The lateral SMAS technique is an approach where a vector of SMASectomy is used to direct the pull of the deep facial fascia. This is different in heavier faces as seen in the figures above because in heavier faces we often remove extra SMAS and defat the fascial layer to improve the overall contour and to reduce the weight on the face. In addition, an extended deep-plane dissection can be performed, which not only describes a bilamellar facelift with completely dissected skin off the fascia, which contributes to more swelling and bruising and possible skin ischemia, but it also allows 2 vectors of movement for the SMAS and the skin.

### Statistics

Patient characteristics and continuous data were analyzed using 2-tailed 2-sample Wilcoxon signed-rank tests at a Type 1 error of 5% (alpha = 0.05), as the continuous data in this study were not normally distributed. Categorical data were evaluated using Chi-squared testing. Data were pooled and organized using Microsoft Excel 2016 (Microsoft, Redmond, WA, USA). Statistics were performed using R-version 4.0.2 Statistical Package (R Core Team, 2013) (IBM, Armonk, NY, USA).^43^

## RESULTS

### Demographics

A total of 241 patients were included in this series, representing 212 (87.97%) female patients and 29 males (12.03%). Demographic information of patients is summarized in Table 1. The mean patient age at the time of surgery was 60.72 ± 8.16 years, with patients ranging from 40 to 85 years old. The average BMI was 23.31 ± 3.49, ranging anywhere from 17.5 to 39.3, although the latter being an outlier. The most common chronic medical condition was hypertension (21.58%). Most of the patients were lifetime nonsmokers, with only 4.15% of patients indicated that they had smoked. These patients were asked to stop smoking 6 weeks before surgery. An additional 28 patients (11.62%) indicated that they “bleed easily,” although no official coagulopathic diagnosis was recorded in the medical record. Steroid use at the time of surgery was reported by 11 patients (4.56%) and hormone replacement therapy use by 26 patients (10.79%). Perioperative stress dose of steroids was administered in these 11 patients.

**Table 1.** Clinical and Demographic Characteristics of All Patients Included in This Series

	Patients			
	No.	% Total	Range	Mean (SD)
Female	212	87.97	—	—
Male	29	12.03	—	—
Age	—	—	40-85	61.3 (±8.2)
BMI^a^	—	—	17.5-39.3	23.5 (±3.49)
Hypertension^b^	52	21.58	—	—
Smoking^c^	10	4.15	—	—
Coagulopathy^d^	28	11.62	—	—
Steroid use^e^	11	4.56	—	—
HRT use^f^	26	10.79	—	—

BMI, body mass index; SD, standard deviation; ^a^One patient record was excluded because patient did not have BMI recorded; ^b^One patient record was excluded because patient did not have the presence or absence of hypertension documented; ^c^Two patient records were excluded because their smoking history was not documented; ^d^Five patient records were excluded because their history of coagulopathy was not documented; ^e^Two patient records were excluded because their medications were not documented; ^f^Two patient records were excluded because their HRT use was not documented; no men were taking HRT.

A total of 117 patients (48.55%) underwent facelift only (including related facial procedures), with 124 patients (51.45%) undergoing an additional procedure. A majority of patients (78.42%) underwent rhytidectomy by SMASectomy technique. Previous facelift had been performed in 81 patients (33.61%). Facelift was supplemented by temple fat injection in 12 patients. Neck liposuction was performed 31.54% of the time. In addition to rhytidectomy, 40 patients underwent suction-assisted lipoplasty of the abdomen, and 27 underwent power-assisted lipoplasty of the abdomen. A large cohort of 118 patients (48.96%) also underwent postoperative laser using Brazilian butt lift (BBL) and halo, BBL alone, BBL and contour, BBL-intense pulsed light (IPL), limelight alone, limelight and BBL, limelight and sciton contour, limelight and halo, limelight and pearl, pearl fractional, sciton contour alone, or venus viva (Sciton, Palo Alto, CA, USA). Laser was used on the face alone, chest alone, neck alone, face and neck, face and chest, face and back, face and hands, or bilateral cheeks. An additional 18 patients underwent CO_2_ laser resurfacing of the face postoperatively.

### Operative Characteristics

The average operating time across all patients in the study was 170.77 ± 76.32 minutes (approximately 2.85 hours), and the average anesthesia time was 194.46 ± 79.21 minutes (Table 2). The difference between operating time and anesthesia time was less than 30 minutes in 96.25% (231/240) of all cases—operative time was not reported in one case. The average operative time of 152.68 ± 51.50 minutes and anesthesia time of 175.00 ± 54.07 minutes were observed among those patients who underwent SMASectomy. This was significantly lower than those who did not undergo SMASectomy, with an average operative time recorded of 265.25 ± 85.25 minutes and anesthesia time of 294.22 ± 85.31 minutes (*P* < 0.000001). Facial drain was placed in 23 cases across all patients (9.54%).

**Table 2.** Operative Characteristics of All Patients Included in This Series

	Patients			
	No.	% Total	Range	Mean (SD)
Facelift				
Alone	117	48.55	—	—
Combined	124	51.45	—	—
Previous facelift	81	33.61	—	—
SMASectomy	189	78.42	—t	—
Neck liposuction	76	31.54	—	—
Facial drain placed	23	9.54	—	—
Operative time (min)	—	—	70-450	170.77 (±76.32)
Anesthesia time (min)	—	—	90-480	194.46 (±79.21)
Fat grafting	51	21.16	—	—
Fat graft volume (mL)	—	—	0.9-20	8.93 (±4.76)
Follow-up time (days)	—		8-5282	239.48 (±614.67)
Postoperative laser	118	48.96	—	—

SMAS, superficial musculoaponeurotic system; SD, standard deviation.

Fat grafting was performed in 51 patients (21.16%). The average graft volume was 8.93 ± 4.76 mL ranging from small volume grafts of 0.9 mL to larger volumes of 20 mL. Follow-up time ranged from 8 days (1 patient) to 176 months, with a mean follow-up time of 7.98 ± 20.49 months. Grafting was done in patients who exhibited temporal hallowing, check involution, prejowl sulcus deepening, or nasolabial deepening and reserved only for thin or atrophic faces.

Although patients underwent additional procedures at the time of the facelift surgery, the mean number of additional procedures in the SMASectomy cohort was 1.16 and 1.38 in the non-SMASectomy cohort (Supplemental Table 1, available online at www.aestheticsurgeryjournal.com). There was no statistical significance to this difference (*P* = 0.27).

### Complications

A total of 32 complications were observed across all patients, with an overall complication rate of 13%. The most common complication was hematoma (5.81%), followed by infection (2.49%) and seroma formation (1.66%). Revision was performed on 38 patients (15.77%). Reason for revision included platysmal banding in the neck, revision of scar tissue around the ear, or for additional reduction of fat from the submental area. No patients developed DVT postoperatively, and no patients experienced any transient or permanent facial nerve injury.

The risk of any complication was not statistically increased with respect to sex, age (less than or greater than the age of 55), BMI (less than or greater than a BMI of 25), smoking status, steroid use, or hormone replacement therapy (HRT) use (Table 3). Although female patients experienced 22 more complications than male patients, the majority of study participants were female; therefore, this increase was not statistically significant (*P* = 0.711). Patients over the age of 55 were also the most represented cohort, with no significant increase in total complications (*P* = 0.556). Although BMI, smoking status, or medication use did not correlate to an increased risk, a positive history of hypertension (defined as a systolic pressure > 140 and diastolic pressure > 90) was associated with an increased risk in any complication (*P* = 0.0444).

**Table 3.** Patient Characteristics and Risk of Complications Across All Patients

	Patients		Complications			
	No.	% Total	No.	% Total	χ² Test	P-Value
Sex					0.137	0.711
Female	212	87.97	29	13.68		
Male	29	12.03	3	10.34		
Age					0.347	0.556
≤55	62	25.73	5	8.06		
>55	179	74.27	27	15.08		
BMI					4.042	0.044
≤25	171	70.95	21	12.28		
>25	70	29.05	11	15.71		
Hypertension					0.239	0.624
Yes	53	21.99	12	22.64		
No	188	78.01	20	10.64		
Smoking					0.098	0.754
Yes	10	4.15	3	30.00		
No	231	95.85	29	12.55		
Steroid use					<0.001	1.000
Yes	11	4.56	2	18.18		
No	230	95.44	30	13.04		
HRT use					0.0266	0.871
Yes	26	10.79	2	7.69		
No	215	89.21	30	13.95		

BMI, body mass index.

Shorter procedure time and anesthesia time (<3 hours) were not associated with a decreased risk of complications. Chi-squared analysis, 1-tailed *t*-test, and 2-tailed *t*-test did not reveal a significant difference (χ² = 0.175, *P* = 0.676). Furthermore, other operative characteristics including history of previous facelift surgery (*P* = 0.343), drain placement (*P* = 1.000), and perioperative or postoperative laser use (*P* = 0.789) did not demonstrate an increased risk of complications (Table 4).

**Table 4.** Operative Characteristics and Risk of Complications in All Patients

	Patients		Complications			
	No.	% Total	No.	% Total	χ² Test	P-value
Operating room time					0.175	0.676
<3 h	153	64.56	23	15.03		
>3 h	84	35.44	10	11.90		
Facelift					0.901	0.343
Previous	66	27.39	11	16.67		
No previous	175	72.61	21	12.00		
Drain placed					<0.001	1.000
Yes	23	9.54	3	13.04		
No	218	90.46	29	13.30		
Postoperative laser					0.072	0.789
Yes	118	48.96	19	16.10		
No	123	51.04	13	10.57		

### Technique

**Figure 5.**
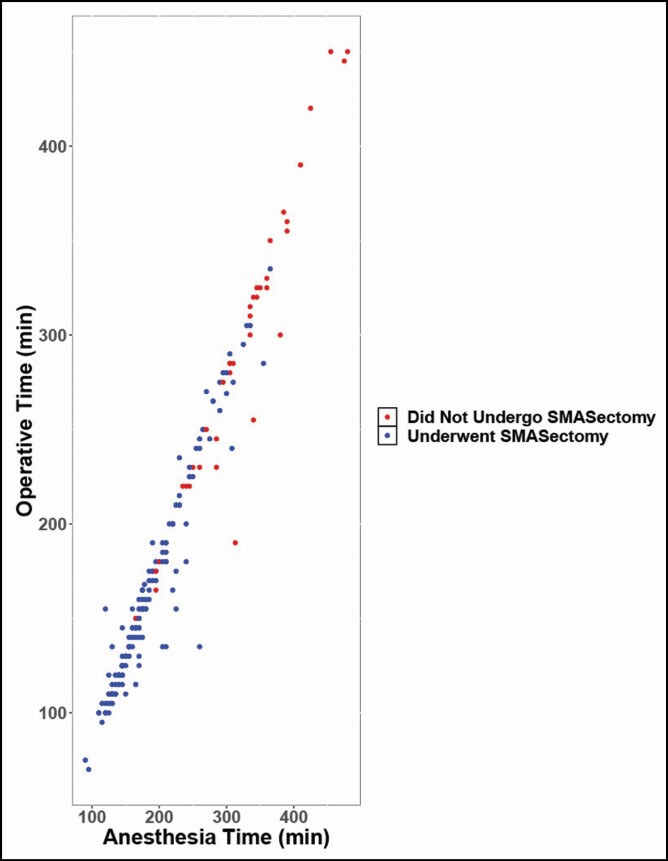
Cluster diagram of operative time and anesthesia time across all 241 patients included in this series, with patients who underwent SMASectomy indicated in blue, and those who did not undergo SMASectomy indicated in red. SMAS, superficial musculoaponeurotic system.

**Figure 6.**
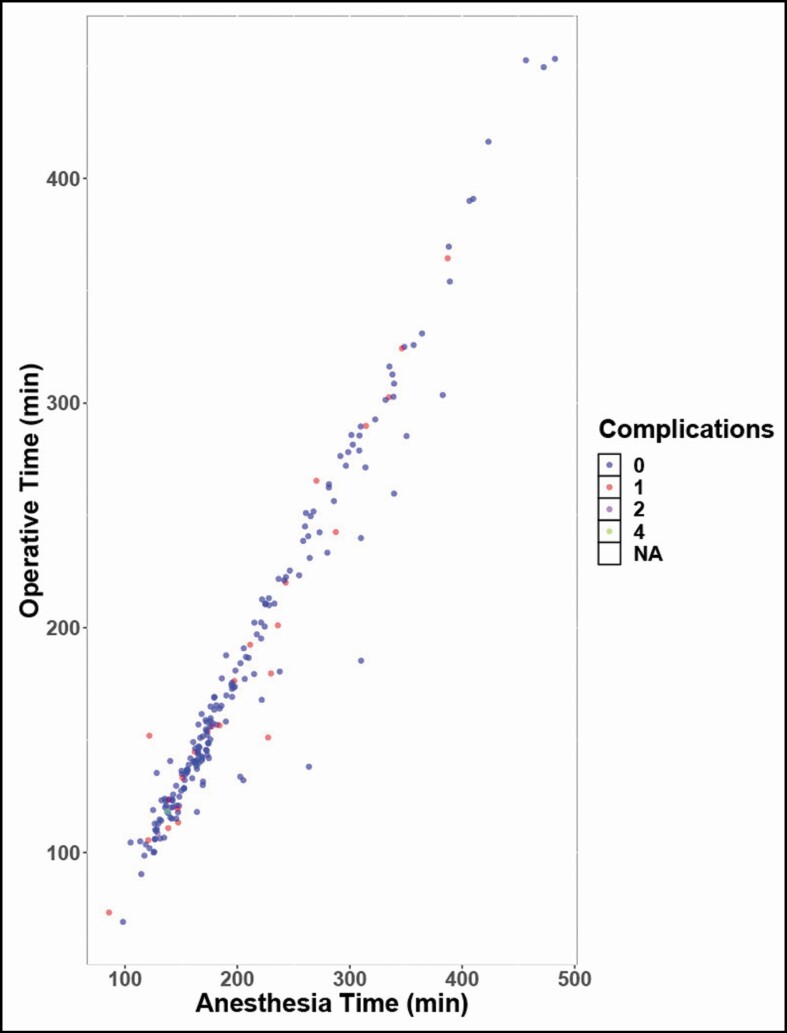
Cluster diagram of operative time and anesthesia time across all patients included in this series relative to the number of complications, with those patients experiencing 0 complications indicated in blue, those with 1 complication in red, 2 complications in purple, and 3 or more complications indicated in green.

A majority of patients (78.42%) underwent SMASectomy at the time of facelift, representing 189 patients. SMASectomy was associated with a significantly lower (*P* < 0.000001), mean procedure time, and mean anesthesia time. Difference in mean anesthesia time was 118.69 minutes, and procedure time was 112.10 minutes (Figure 5). There were no observed facial nerve injuries among patients who underwent SMASectomy. Furthermore, this technique was not associated with a significantly increased overall rate of complications (*P* = 0.602). Complications among those patients who underwent SMASectomy and those who did not are summarized in Table 5. Although a greater number of complications were observed in the SMASectomy cohort, there was no statistically significant difference in the most commonly observed complications, including hematoma (*P* = 0.680), seroma (*P* = 1.000), flap necrosis (*P* = 1.000), and infection (*P* = 0.920). SMASectomy was associated with an increased revision rate (17.98%) compared with all other patients (7.69%); however, this was also not statistically significant (*P* = 0.940). In addition, few patients experienced more than one complication, independent of technique (Figure 6).

**Table 5.** Relative Risk of Complications and Operative Characteristics When Performing SMASectomy

	Anesthesia time (min)			Procedure time (min)			
	Range	Mean (SD)	P-value	Range	Mean (SD)	P-value	
SMASectomy			<0.00001			<0.00001	
Yes	90-365	175.53 (±54.07)		70-335	153.15 (±51.50)		
No	125-480	294.22 (±85.31)		110-450	265.25 (±85.25)		
Complication	Transient FN injury	Permanent FN injury	Hematoma	Seroma	Flap necrosis	Infection	Revision
Total No.							
SMASectomy	0	0	11	4	2	6	34
No SMASectomy	0	0	3	0	0	0	4
*P*-value	—	—	0.068	1.00	1.00	0.92	0.94
Relative risk	—	—	0.15	—	—	—	0.81

FN, facial nerve; SD, standard deviation; SMAS, superficial musculoaponeurotic system.

**Figure 7.**
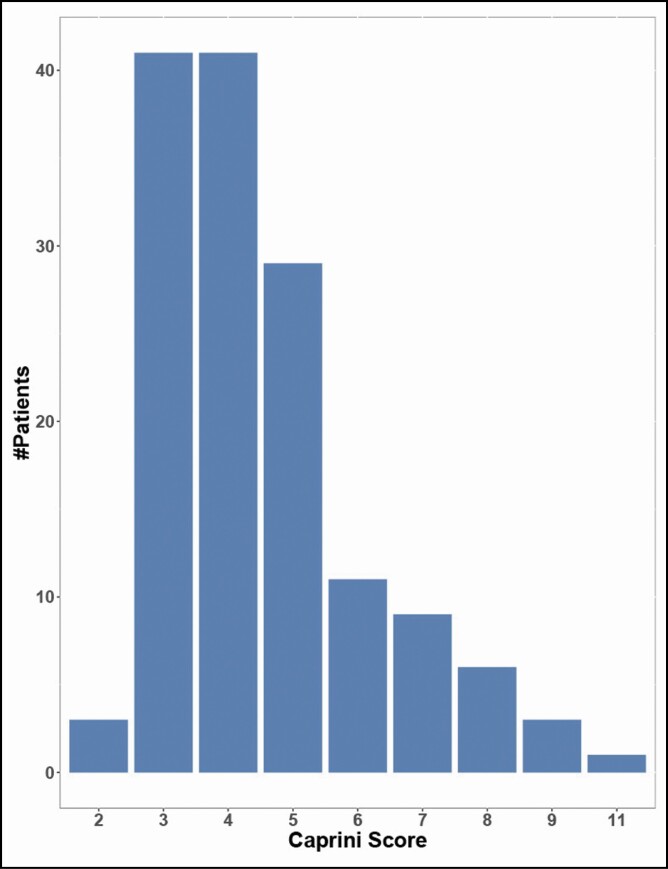
Caprini scores of all included patients and corresponding categorical risk of thromboembolic events.

Caprini scores were calculated for 148 patients (61.41%), with a mean score of 4.28 ± 1.62, representing an average moderate risk of DVT among these patients (Figure 7). No DVT events were observed in this patient population.

## DISCUSSION

Surgical and nonoperative facial rejuvenation remains one of the most common cosmetic procedures performed in the United States despite recent decreases in overall procedures performed by plastic surgeons.^1^ This decline is likely multifactorial but may also be attributed to the larger number of otolaryngologic surgeons now performing these procedures.^34^ The public perception of unnatural outcomes as well as potentially morbid complications such as DVT and facial nerve injury may have also had an influence on the individual decision to undergo rhytidectomy.

**Figure 8.**
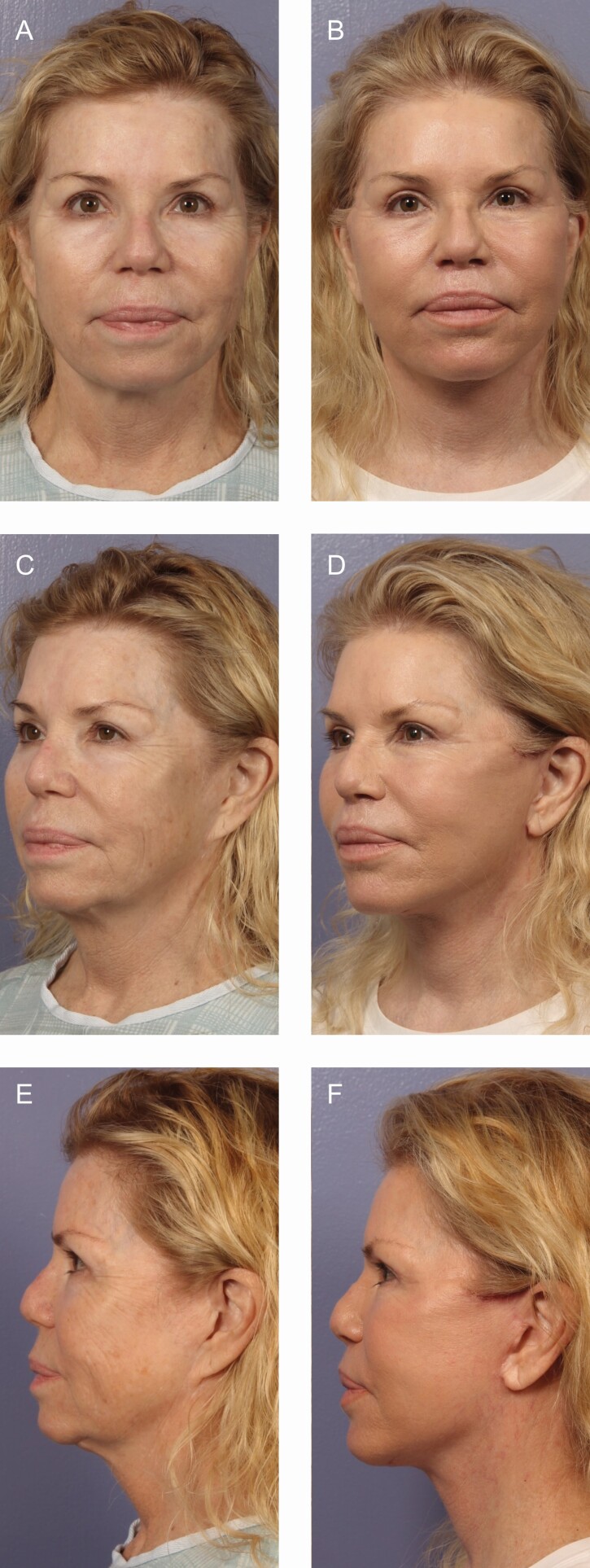
A 62-year-old female lateral SMASectomy with SMAS flap and upper blepharoplasty with postoperative halo laser resurfacing (A, B) frontal view, (C, D) oblique view, and (E, F) lateral view with 6 months follow-up. SMAS, superficial musculoaponeurotic system.

**Figure 9.**
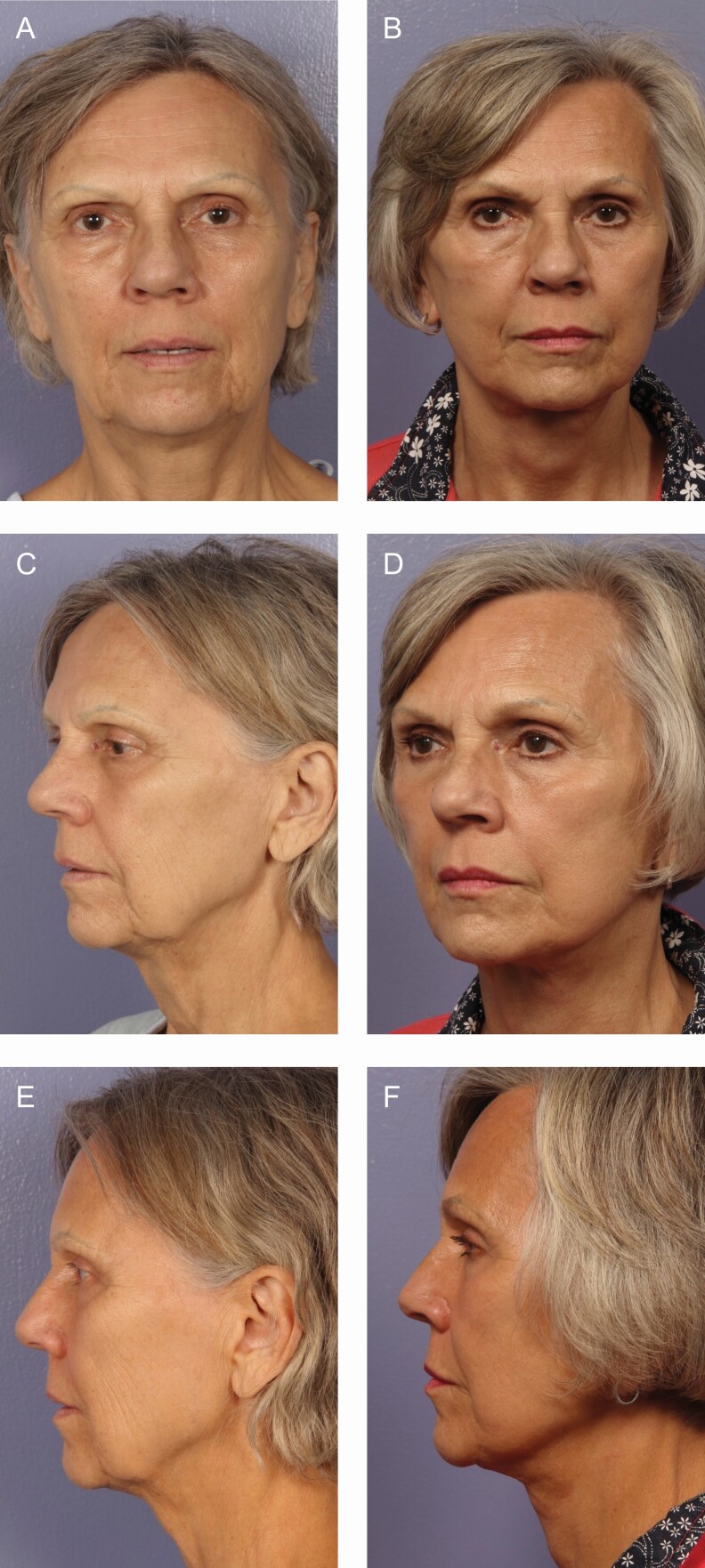
A 70-year-old female deep-plane SMASectomy with upper blepharoplasty brow lift with additional halo and BBL postoperative laser contouring (A, B) frontal view, (C, D) oblique view, and (E, F) lateral view with 6 months follow-up. SMAS, superficial musculoaponeurotic system.

**Figure 10.**
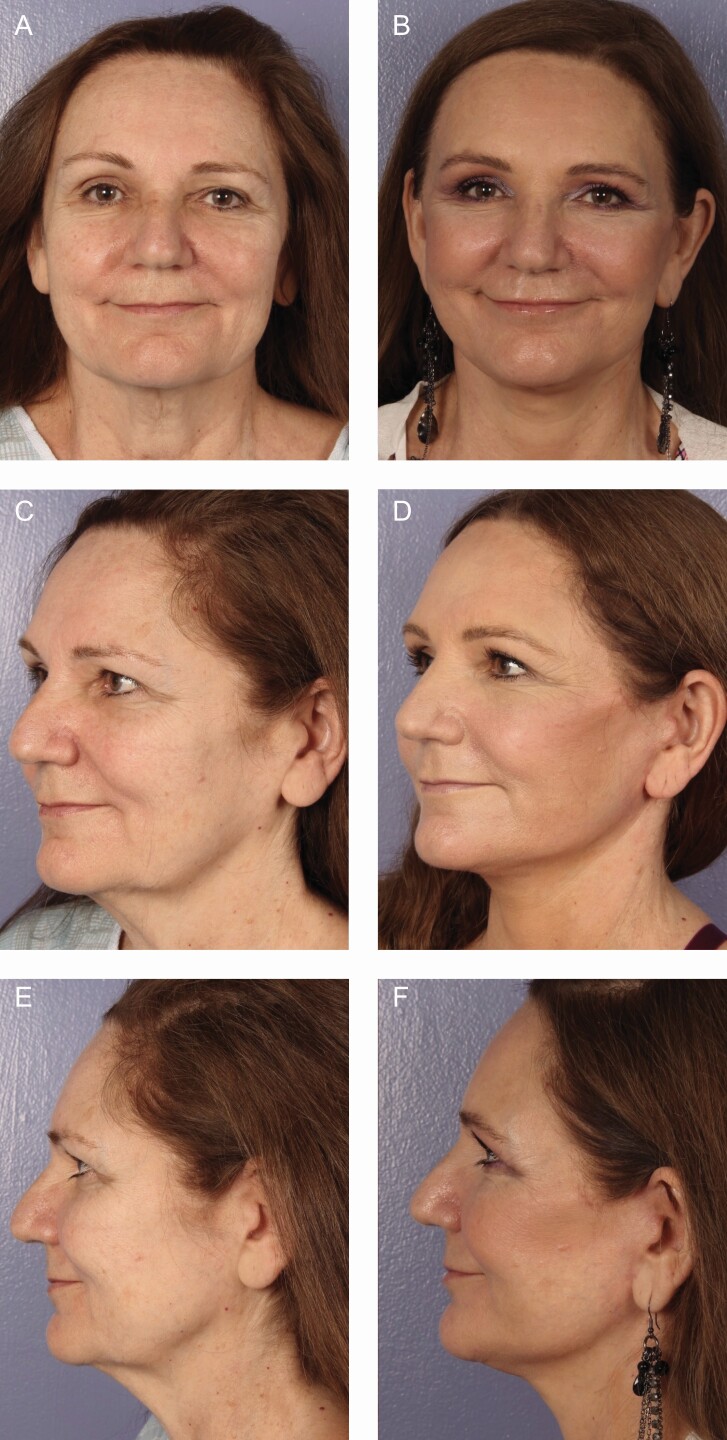
A 69-year-old female extended deep-plane SMASectomy with forehead lift, bilateral upper and lower blepharoplasty, and fat grafting to the face with additional halo and BBL laser treatment postoperatively (A, B) frontal view, (C, D) oblique view, and (E, F) lateral view with 12 months follow-up. SMAS, superficial musculoaponeurotic system.

There is a plethora of various techniques for performing rhytidectomy, and in this study, SMASectomy is used in the large majority of patients. SMASectomy is often criticized by many for its added risk of facial nerve injury although it is noted by some to improve cosmesis and longevity of results.^35^ SMASectomy also accomplishes the goal of a facelift by putting tension on the face but avoids the need for an extended SMAS dissection or deep-plane dissection, leading to excellent results. In this study, we examine our series of patients over the past 15 years in order to determine the differences in complications rates between SMASectomy and other facelift techniques as well as to identify and discuss what we believe to be the significant advantages to SMASectomy other than the cosmetic outcomes previously discussed. Examples provided include the lateral SMASectomy technique (Figure 8), deep-plane SMASectomy technique (Figure 9), and extended deep-plane SMASectomy technique (Figure 10).

The added risk of venous thromboembolism (VTE) in the setting of facelift surgery has recently been documented and discussed in the literature.^3,29,31,36^ In our series of patients, we found that a reduction in operative time and anesthesia time may entirely eliminate this added risk, even with a mean Caprini score representing moderate risk among most of the patients in our study. Other than compression stockings and serial compression devices, no other prophylactic measures were taken. Despite this, we observed no embolic events in our facelift population, likely due to significant decreases in operative and anesthesia time as a result of the SMASectomy technique. In comparison, studies by Abboushi et al and Reinisch et al have demonstrated DVT incidence of 0.3% and 0.35%, respectively.^29,36^ In addition, Abboushi et al reported a mean operative time of 255.6 minutes, compared with 170.8 minutes in this series across all patients, and an even shorter mean operative time of 112.1 minutes in patients that underwent SMASectomy.^29^ Our decrease in DVT is not simply due to patient selection as our Caprini scoring demonstrates a variety of different risk profiles.

There is a well-documented increased VTE risk with longer anesthesia and operative times.^37-41^ The SMASectomy technique directly reduces the time spent on the table and thus may confer a significantly decreased VTE risk. Furthermore, SMASectomy offers a better aesthetic technique and, as demonstrated in this study, when performed correctly does not increase the risk of facial nerve injury or any other complication common among patients undergoing a facelift.

There are many prophylactic interventions the surgeon may undertake in reducing the risk of thromboemoblic events; the authors believe that reducing operative time and maximizing operative efficiency are some of the most effective.^42^ The results of this study demonstrate that SMASectomy technique results in a significantly lower operative time and anesthesia time, without any significant increase in the rate of adverse events, and should be the preferred technique for rhytidectomy.

There were several limitations in our assessment. Additional procedures performed concomitantly with the facelift procedure may confound our operative time data. The study may also not include enough patients to demonstrate a significantly different rate of thromboembolic events in this set of patients, as the rate reported in the literature is extremely low. Even so, the data herein demonstrate that SMASectomy is a safe approach with no significant difference in complications and revision rates in comparison to standard techniques such as plication, which may confer a reduced risk of DVT.

## CONCLUSIONS

In the hands of an experienced surgeon, SMASectomy facelift technique may reduce the risk of embolic events during rhytidectomy secondary to the decrease in operating time and anesthesia time and also provide high-quality results. The authors recommend that SMASectomy be the primary facelift technique offered to patients both for this reduced risk and for the excellent aesthetic outcomes well documented in the literature. Furthermore, as demonstrated in this series, the potential risk of facial nerve injury can be completely mitigated if performed by a well-trained surgeon with careful dissection and awareness of local anatomy.

## Supplementary Material

ojab030_suppl_Supplementary_Materials
