# Supplementary material for: Evaluating Facelift Complications and the Effectiveness of the SMASectomy Technique: A Single Center’s 15-Year Experience
Source: Aesthet Surg J Open Forum. 2021 Aug 20;3(4):ojab030. doi: 10.1093/asjof/ojab030 (PMC8489308; doi:10.1093/asjof/ojab030)
Supplement: ojab030_suppl_Supplementary_Materials [file ojab030_suppl_supplementary_materials.docx]

**Supplemental Table 1**. Additional Procedures in Patients With and Without SMASectomy Facelift Procedure

|  | SMASectomy | Non-SMASectomy | Total |
| --- | --- | --- | --- |
| Plication | 1 | 42 | 43 |
| Neck liposuction | 53 | 23 | 76 |
| SMAS flap | 0 | 5 | 5 |
| Platysma plication | 8 | 13 | 21 |
| Face fat grafting | 21 | 29 | 50 |
| Additional face procedure |  |  |  |
| Lip lift | 0 | 1 | 1 |
| Brow lift | 12 | 5 | 17 |
| Upper bleph | 63 | 17 | 80 |
| Lower bleph | 56 | 19 | 75 |
| Other | 91 | 32 | 123 |
